# Laparoscopic Abdominoperineal Resection for Ischemic Colitis after Laparoscopic Partial Resection of the Descending Colon: Case Report

**DOI:** 10.70352/scrj.cr.25-0572

**Published:** 2026-01-31

**Authors:** Mitsuki Yokota, Hidekazu Takahashi, Asako Mike, Kei Fukumori, Yuka Iwami, Juavijitjan Watsapol, Satoshi Ishikawa, Shohei Takaichi, Masakatsu Paku, Kazuya Iwamoto, Tomofumi Ohashi, Yujiro Nakahara, Kohei Murakami, Tadafumi Asaoka, Ichiro Takemasa, Takeshi Omori

**Affiliations:** 1 Department of Gastroenterological Surgery, Osaka Police Hospital, Osaka, Osaka, Japan; 2 Department of Pathology, Osaka Police Hospital, Osaka, Osaka, Japan

**Keywords:** partial resection of the descending colon, ischemic colitis, laparoscopic abdominoperineal resection

## Abstract

**INTRODUCTION:**

With advances in laparoscopic surgery, more sophisticated vessel-preserving techniques have become standardized. Laparoscopic partial colectomy aimed at maximizing colonic preservation is now widely performed. Along with this trend, cases of ischemic colitis developing after colorectal cancer surgery have occasionally been reported; however, cases requiring surgical resection remain exceedingly rare. Here, we report a case of ischemic colitis that developed 2 years and 2 months after partial laparoscopic resection of the descending colon, necessitating laparoscopic abdominoperineal resection.

**CASE PRESENTATION:**

A 65-year-old male underwent laparoscopic partial resection of the descending colon with preservation of the superior rectal artery to treat descending colon cancer at the age of 62 years. Two years and 2 months postoperatively, the patient developed left abdominal pain. Contrast-enhanced CT and colonoscopy revealed ischemic colitis. Because conservative management was ineffective, surgical resection was required, and laparoscopic abdominoperineal resection was performed. Histopathological examination confirmed a diagnosis of ischemic colitis. The patient was discharged 48 days after surgery.

**CONCLUSIONS:**

Ischemic colitis occurring after colorectal cancer surgery is rare, and surgical intervention is extremely uncommon in such cases. Here, we present this case with a review of the relevant literature.

## Abbreviations

CE
: contrast enhanced
CS
: colonoscopy
EVG
: Elastica–van Gieson
H&E
: hematoxylin and eosin
IC
: ischemic colitis
IMA
: inferior mesenteric artery
LAPR
: laparoscopic abdominoperineal resection
LCA
: left colic artery
SRA
: superior rectal artery

## INTRODUCTION

IC after colorectal cancer surgery is rare, and cases that require surgical resection are even rarer. Herein, we report a case of IC that developed 2 years and 2 months after partial resection of the descending colon, which was treated with LAPR, along with a review of the relevant literature.

## CASE PRESENTATION

A 65-year-old male with a history of gastroduodenal ulcers underwent laparoscopic partial resection of the descending colon with D2 lymph node dissection at age 62 for descending colon cancer. The IMA was preserved, and the LCA was divided at its origin. The inferior mesenteric vein was also divided at the same level, while the SRA was preserved. A functional end-to-end anastomosis was performed extracorporeally.

Histopathological findings revealed type 0-IIa+IIc, 10 mm, adenocarcinoma (tub1 >> tub2), pT2, INFb, Ly0, V0, Pn0, pPM0, pDM0, pRM0, n(0/15), and pStage I.

**Fig. 1:**
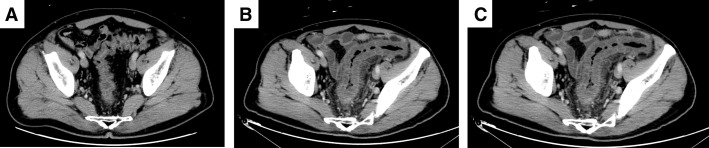
Contrast-enhanced abdominal CT findings. (**A**) Twenty-one months after the initial surgery. The distal colon adjacent to the anastomosis shows no obvious abnormal findings. (**B**) Twenty-six months after the initial surgery. The distal colon near the anastomosis demonstrates circumferential bowel wall thickening accompanied by increased attenuation of the surrounding pericolic fat, suggestive of inflammatory changes. (**C**) Twenty-seven months after the initial surgery. Follow-up imaging reveals no appreciable improvement in bowel wall thickening or pericolic fat stranding in the distal colon adjacent to the anastomosis.

**Fig. 2:**
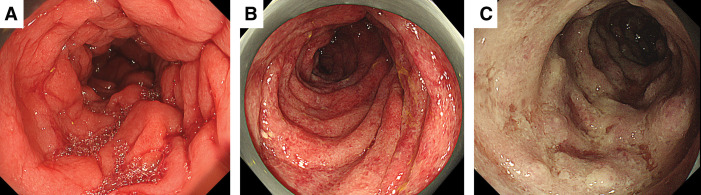
Colonoscopy findings. (**A**) Twenty-six months after the initial surgery. Diffuse mucosal erythema and edematous changes are observed from the sigmoid colon to the rectum. (**B**) Twenty-seven months after the initial surgery. The distal bowel beyond the anastomosis demonstrates progression of mucosal erythema and edema compared with the previous examination. (**C**) Twenty-eight months after the initial surgery. Marked edematous changes and ischemic findings are noted predominantly in the sigmoid colon, extending throughout the distal colon beyond the anastomosis.

The patient’s postoperative course was uneventful, and no recurrence was observed. However, 26 months after the initial surgery, the patient developed left abdominal pain accompanied by severe anal pain. CE-CT (**Fig. 1B**) and CS (**Fig. 2A**) revealed inflammatory changes distal to the site of anastomosis. Notably, CE-CT performed 21 months after the initial surgery showed no abnormal findings in the same region (**Fig. 1A**).

Twenty-seven months after the initial surgery, hematochezia developed, and the patient was admitted for further evaluation and treatment. On admission, the patient was alert and hemodynamically stable; however, the C-reactive protein level was elevated to 2.3 mg/dL. Repeat CE-CT showed persistent inflammatory changes distal to the anastomosis (**Fig. 1C**), and CS demonstrated progression of the inflammatory findings extending from the anastomosis to the anus (**Fig. 2B**).

**Fig. 3:**
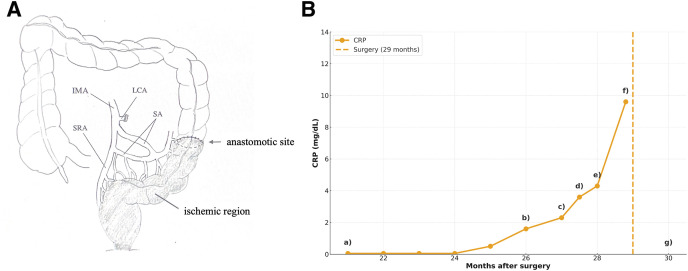
(**A**) Ischemic region. Illustration demonstrating the distribution of intestinal ischemia identified in this case. (**B**) Clinical course and CRP trend after the initial surgery. (a) CE-CT showed no abnormality (**Fig. 1A**); CRP 0.05 mg/dL. (b) Onset. CE-CT and CS revealed inflammation distal to the anastomosis (**Figs. 1B** and **2A**); CRP 1.6 mg/dL. (c) Hospitalization. Inflammation worsened on CE-CT and CS (**Figs. 1C** and **2B**). Conservative therapy started; CRP 2.3 mg/dL. (d) CRP increased to 3.6 mg/dL. 5-ASA and budesonide was initiated. (e) Further exacerbation on colonoscopy (**Fig. 2C**). Diagnosed as ischemic colitis; CRP 4.3 mg/dL. (f) Preoperative CRP peaked at 9.6 mg/dL. (g) Postoperative CRP returned to baseline (0.05 mg/dL). 5-ASA, 5-aminosalicylic acid; CE, contrast enhanced; CRP, C-reactive protein; CS, colonoscopy; IMA, inferior mesenteric artery; LCA, left colic artery; SA, sigmoid artery; SRA, superior rectal artery

Considering inflammatory bowel disease as a differential diagnosis, conservative management, including fasting, was initiated along with 5-aminosalicylic acid and budesonide as empirical therapy. However, a CS performed 28 months after the initial surgery showed further exacerbation of inflammation (**Fig. 2C**), leading to a definitive diagnosis of IC. The ischemic areas are shown in **Fig. 3A**.

In the present case, conservative management was continued; however, the patient showed no meaningful improvement over time. Because the ischemic segment extended to the vicinity of the anal canal, any surgical intervention was expected to markedly impair anorectal function and consequently reduce QOL. Therefore, establishing a definitive diagnosis and determining whether the ischemic changes were irreversible required careful and repeated evaluation, which inevitably prolonged the decision-making process.

Approximately 3 months after the onset of IC (29 months after the initial surgery), the patient ultimately elected not to preserve the anus due to persistent and severe anal pain. At that time, 2 surgical options were considered: a 2-stage ultralow anterior resection with diverting ileostomy or LAPR. Given the patient’s strong preference and the anticipated difficulty in preserving acceptable anorectal function, LAPR was selected. The treatment course is illustrated in **Fig. 3B**.

**Fig. 4:**
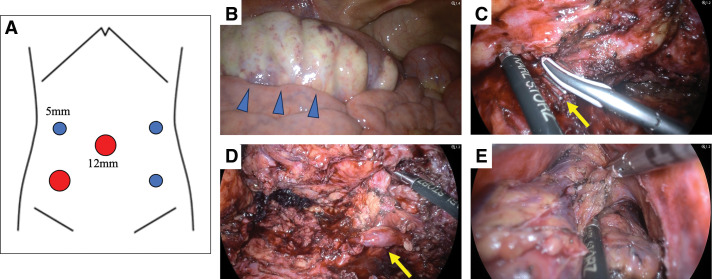
Intraoperative findings. (**A**) Five-port placement. (**B**) The sigmoid colon showed ischemic changes extending to the serosal surface. (**C**) Division of IMA. (**D**) The left ureter was firmly adherent to the mesentery. (**E**) The dissection plane of the rectum from the transabdominal approach was connected with that from the transperineal approach. IMA, inferior mesenteric artery

The patient was placed in the lithotomy position and LAPR was performed using 5 ports. The bowel distal to the transverse colon was dilated with ischemic changes, particularly evident on the serosal surface of the sigmoid colon. After mobilization of the splenic flexure, a medial approach was used, and the IMA was divided. A lateral approach was also used, during which the left ureter was firmly adhered to the mesentery and carefully dissected. Circumferential dissection of the rectum was performed, and the peritoneal reflection was incised to allow maximal distal dissection around the rectum. Transanal total mesorectal excision was combined: the perianal skin was incised, and the dissection plane was connected to the intra-abdominal approach, enabling removal of the rectum and anus en bloc. The transverse colon was divided extracorporeally and a transverse colostomy was created in the left upper abdomen. The operation time was 9 h and 38 min, and the estimated blood loss, including ascites, was 800 mL (**Fig. 4**).

**Fig. 5:**
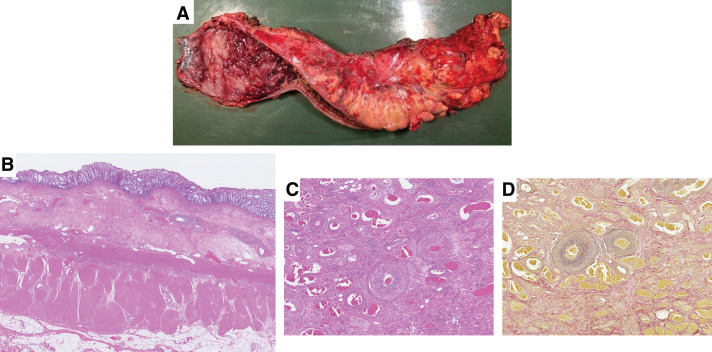
Histopathological findings. (**A**) The resected specimen showed diffuse mucosal sloughing with a dark reddish discoloration extending from the anastomotic site to the anal verge. (**B**) Extensive mucosal fibrin deposition, erosion with epithelial necrosis, and ulcer formation were observed throughout the bowel. In the submucosal layer, fibroblastic proliferation, vascular proliferation, and narrowing or collapse of vascular lumina were noted, while the muscularis propria was largely preserved. In the subserosal layer, widespread fat degeneration was evident, accompanied by inflammatory cell infiltration, granulomatous changes, fibrous proliferation, and hyalinized fibrosis. These findings were considered consistent with ischemic colitis associated with relatively prolonged ischemia. (**C**) The veins are dilated with prominent intraluminal accumulation of blood cells. (**D**) EVG staining showing veins filled with intraluminal blood cells. EVG, Elastica–van Gieson

Gross examination of the resected specimen revealed diffuse mucosal sloughing and dark reddish discoloration extending from the anastomosis to the anal verge (**Fig. 5A**). Histologically, H&E staining showed fibrin exudation, erosion, and ulcer formation with epithelial necrosis across an extensive area of the mucosa. Submucosal findings included fibrosis, neovascularization, and vascular narrowing or collapse, whereas the muscularis propria was largely preserved. Subserosal findings showed widespread fat degeneration, inflammatory cell infiltration, granulomatous changes, connective tissue proliferation, and hyalinized fibrosis consistent with IC associated with relatively prolonged ischemia (**Fig. 5B**).

Furthermore, on both H&E staining (**Fig. 5C**) and EVG staining (**Fig. 5D**), dilated veins with marked intraluminal pooling of blood cells were observed, which is indicative of venous congestion.

Oral intake was resumed on POD 13. Although perineal wound healing required time, the patient was discharged independently on POD 48. One year after the surgery, the patient remained free of recurrence, metastasis, and abdominal symptoms, maintaining a favorable clinical course.

## DISCUSSION

IC develops because of an insufficient blood supply to the intestine. In patients who have undergone colorectal cancer surgery, intraoperative or postoperative vascular injury, hemodynamic alterations, and postoperative scarring can contribute to an increased risk of IC.^1,2)^ Postoperative IC typically occurs relatively early, usually within 6 months after surgery, and in many cases, is confined to the mucosa or submucosa, presenting as a mild form of the disease. Such cases generally respond well to conservative management, including fasting, fluid resuscitation, and antibiotic therapy.^3,4)^

In contrast, IC that develops as late as 26 months postoperatively, as in our case, is extremely rare. To clarify the rarity of such cases, we performed a PubMed search of literature published between 2014 and 2024 using combinations of the terms “colorectal cancer,” “IC,” and “case report.” Only English-language case reports on IC after colorectal cancer surgery were included.

This search identified only 12 previously reported cases, of which only 2 required surgical resection (**Table 1**). Among the 10 cases that improved with conservative therapy, the mean duration to recovery was 4.2 months, and the median duration was 7.3 months. In the case reported by Fujii et al.^5)^, a 60-year-old man initially underwent conservative treatment; however, due to insufficient improvement, a temporary ileostomy was created approximately 30 days after treatment initiation. Persistent stenosis of the ischemic bowel segment was observed over the following 3 months, and the patient ultimately required laparoscopic low anterior resection to remove the stenotic segment, including the anastomotic site. Our case is therefore notable not only for the unusually long interval between colorectal cancer surgery and the onset of IC but also for the severity of the disease, as conservative management was unsuccessful and surgical resection was required.^6,7)^

**Table 1:** Ischemic colitis after colorectal cancer surgery

Publication year	Author	Age	Sex	Surgical procedure for colorectal cancer	Interval to onset of ischemic colitis	Ischemic site	Treatment for ischemic colitis	Outcome
2015	Lim et al.^20)^	57	Male	Robot-assisted ultra-low anterior resection	4 days	Distal to the anastomosis	Conservative management	Improved
2015	Lim et al.^20)^	54	Female	Robot-assisted ultra-low anterior resection	5 days	Proximal to the anastomosis	Conservative management	Improved
2015	Lim et al.^20)^	63	Male	Robot-assisted ultra-low anterior resection	2 days, 6 months	Distal to the anastomosis	Conservative management	Improved
2015	Lim et al.^20)^	60	Male	Robot-assisted ultra-low anterior resection	1 day, 14 days	Proximal to the anastomosis	Conservative management	Improved
2018	Kamada et al.^21)^	80	Male	Laparoscopic left hemicolectomy (SRA preserved)	4 months	Distal to the anastomosis	Conservative management	Improved
2020	Fujii et al.^5)^	75	Female	Laparoscopic left hemicolectomy (SRA preserved)	19 months	Distal to the anastomosis~rectum	Conservative management	Improved
2020	Fujii et al.^5)^	57	Male	Laparoscopic left hemicolectomy (SRA preserved)	6 months	Distal to the anastomosis~rectum	Conservative management	Improved
2020	Fujii et al.^5)^	67	Male	Laparoscopic left hemicolectomy (SRA preserved)	34 months	Distal to the anastomosis~rectum	Conservative management	Improved
2020	Fujii et al.^5)^	60	Male	Laparoscopic left hemicolectomy (SRA preserved)	5 months	Distal to the anastomosis~rectum	Laparoscopic low anterior resection	Improved
2021	Yoshida et al.^22)^	60	Male	Laparoscopic sigmoidectomy	6 months	Rectum	Conservative management	Improved
2024	Lee and Park^9)^	68	Female	Laparoscopic sigmoidectomy	12 months	Descending colon	Conservative management	Improved
2024	Ishimaru et al.^11)^	73	Male	Laparoscopic sigmoidectomy (IMA preserved)	11 months	Distal to the anastomosis/rectum	Laparoscopic abdominoperineal resection	Improved
	Present case	65	Male	Laparoscopic left hemicolectomy (IMA/SRA preserved)	24 months	Distal to the anastomosis~rectum	Laparoscopic abdominoperineal resection	Improved

Most cases have been successfully managed with conservative treatment.

IMA, inferior mesenteric artery; SRA, superior rectal artery

Recent studies have highlighted postoperative venous congestion as a potential contributor to IC.^8,9)^ In cases where the inferior mesenteric vein is ligated at a high level during left-sided colectomy or low anterior resection, impaired venous drainage of the remnant colon may elevate local venous pressure and cause congestion.^10)^ Such venous outflow disturbances may lead to capillary stasis, mucosal edema, hemorrhagic changes, and ultimately ischemic injury.^8,11)^

In the present case, the SRA was preserved, which may have been protective from an arterial standpoint. However, the inferior mesenteric vein was divided distal to the resection line, and venous congestion may have occurred in the sigmoid colon region. Histopathological examination also revealed findings compatible with venous congestion, suggesting that venous stasis likely contributed to the development of IC.

Possible mechanisms for late-onset IC include chronic bowel traction and local blood flow disturbances resulting from postoperative adhesions and scarring. In addition, vascular risk factors such as hypertension, diabetes, and atherosclerosis may act synergistically, leading to progressive impairment of the intestinal microcirculation.^12,13)^ Furthermore, chronic inflammation can induce perivascular fibrosis and intimal thickening, gradually compromising blood flow until a threshold is crossed, and ischemic changes manifest abruptly.^14)^

In severe cases, timely surgical intervention before the development of intestinal necrosis or perforation is essential to improve prognosis.^15–17)^ A delayed diagnosis can directly lead to sepsis and multiple organ failure. Therefore, clinicians should recognize that IC may develop not only in the early postoperative period but also several years after surgery. Persistent or recurrent nonspecific symptoms, such as abdominal pain, hematochezia, and fever, should be promptly evaluated with CE-CT or other imaging modalities, and surgical intervention should be considered if signs of severe disease are present.^18,19)^

Our case is exceptionally rare in terms of both the delayed onset and severity of IC following colorectal cancer surgery. The accumulation and analysis of similar cases may help to clarify the pathophysiology of late-onset postoperative IC, identify risk factors, and establish preventive strategies.

## CONCLUSIONS

Here, we report an extremely rare case of IC that developed in the late postoperative period after colorectal cancer surgery and required surgical resection. The accumulation of similar cases may contribute to a better understanding of the disease mechanisms and support the development of preventive strategies for postoperative patients.
